# The Diverse Network of Brain Histamine in Feeding: Dissect its Functions in a Circuit-Specific Way

**DOI:** 10.2174/1570159X21666221117153755

**Published:** 2023-03-22

**Authors:** Lingyu Xu, Wenkai Lin, Yanrong Zheng, Yi Wang, Zhong Chen

**Affiliations:** 1 Institute of Pharmacology & Toxicology, College of Pharmaceutical Sciences, Zhejiang University, Hangzhou, China;; 2 Key Laboratory of Neuropharmacology and Translational Medicine of Zhejiang Province, School of Pharmaceutical Sciences, Zhejiang Chinese Medical University, Hangzhou, China

**Keywords:** Histamine, histamine receptor ligands, neural circuit, feeding, drug target, brain histaminergic network

## Abstract

Feeding is an intrinsic and important behavior regulated by complex molecular, cellular and circuit-level mechanisms, one of which is the brain histaminergic network. In the past decades, many studies have provided a foundation of knowledge about the relationship between feeding and histamine receptors, which are deemed to have therapeutic potential but are not successful in treating feeding-related diseases. Indeed, the histaminergic circuits underlying feeding are poorly understood and characterized. This review describes current knowledge of histamine in feeding at the receptor level. Further, we provide insight into putative histamine-involved feeding circuits based on the classic feeding circuits. Understanding the histaminergic network in a circuit-specific way may be therapeutically relevant for increasing the drug specificity and precise treatment in feeding-related diseases.

## 1 INTRODUCTION

Central histamine, secreted by a small group of neurons confined to the hypothalamic tuberomammillary nucleus (TMN), acts as a modulatory neurotransmitter through G protein-coupled receptors and fine-tunes the action of other neurotransmitters on target neurons in the mammalian brain. Apart from the classical synaptic transmission, histamine also diffuses through large areas of the nervous system *via* volume transmission and affects diverse populations of neurons [1]. Nevertheless, the histaminergic system has received considerably less attention than other neurotransmitters such as dopamine, noradrenaline and serotonin for its moderate functions and late discovery [2]. The past decade has, or past few decades have witnessed an expansion of knowledge in histamine neurobiology for its close relation with many physiological functions like feeding, circadian rhythms, cognition, locomotion, and pathological diseases like multiple sclerosis, Parkinson’s disease, bulimia nervosa and anorexia nervosa [3-9]. Thus, understanding the precise roles of brain histamine in these functions is crucial for comprehending the underlying mechanisms and further revealing promising therapeutic targets.

Among the aforementioned functions related to brain histamine activity, feeding behavior has gained much interest due to the increasing concern of obesity globally. Eating disorders like anorexia nervosa, bulimia, and binge-eating syndrome have been very common in the clinical setting recently, yet they are very difficult to be treated owing to the lack of effective drugs and their complex mechanisms. Drugs for long-term treatment of obesity, such as sibutramine (a monoamine reuptake inhibitor) and orlistat (a peripherally acting lipase inhibitor), working by different mechanisms, have also reflected the complex etiology of the disease [10, 11]. Moreover, long-term use of anti-obesity drugs, such as clocasserin, phentermine-topiramate and naltrexone-bupropion combinations, can affect mood and cognitive function, leading to psychiatric problems such as anxiety, depression and drug dependence [12, 13]. Thus, more novel therapeutic strategies, new drug discovery and development, and precise treatment are urgently needed to improve unsatisfactory pharmacotherapy for feeding-related diseases.

Multiple studies have looked into the relationships between histamine and feeding behaviors, and efforts have been paid to examine the therapeutic potential of histamine receptor ligands for the treatment of weight problems, despite some contradictory results (discussed below). In this review, we shed light on the histaminergic network from a circuit-specific aspect in feeding behavior, which may help to interpret some contradictory results of previous preclinical studies and give possible research insights to better understand how the histaminergic system functions in feeding.

We systematically searched PubMed for publications in English with the combined keywords, including “histamine”, “histaminergic”, “feeding”, “circuit”, “food intake”, “histamine receptor”, and “drug target”. Reference lists of relevant papers were also checked for additional studies. We arbitrarily chose seminal work and clinical studies with the highest level of evidence. We have also used some earlier articles and reviews pertinent to the discussion.

## 2 PROJECTIONS OF HISTAMINERGIC NEURONS AND DISTRIBUTION OF ITS RECEPTORS

The cell bodies of histaminergic neurons are conscribed to the hypothalamic TMN and send their fiber projections to innervate nearly the entire central nervous system (CNS). Histamine is synthesized from the precursor amino acid histidine by histidine-decarboxylase (HDC) and stored in cell somata, which is carried into vesicles through the vesicular monoamine transporter (VMAT-2) and released to bind post-synaptic or pre-synaptic histamine receptors. It is eventually methylated into tele-methylhistamine by histamine methyltransferase after being released into extracellular space [8, 14, 15]. Four types of G protein-coupled receptors interacting with histamine as ligands have been identified so far, H1R, H2R, H3R and H4R, respectively [16-19].

H1Rs are located post-synaptically and found throughout the CNS with various densities that do not always match the histaminergic innervation. Studies using [^3^H] mepyramine binding as an indicator showed that high densities of H1Rs are found in brain regions associated with cognition, consciousness, sleep, feeding and behavioral state control like the hypothalamus, aminergic and cholinergic brainstem nuclei, thalamus and cortex [15, 20, 21], which are functionally matched with the histaminergic system. Histamine can activate neurons in multiple brain regions, including the brain stem, hypothalamus, thalamus, amygdala, septum, hippocampus, and cortex [22-29], by binding to H1Rs, which couple to G*q* proteins.

H2Rs are also located post-synaptically. Although sharing high similarities in the brain region distribution of H1Rs and H2Rs, the distribution of H2Rs in the rodent brain is more consistent with histaminergic projections than H1Rs, suggesting that H2Rs may mediate much more postsynaptic actions of neuronal histamine [30, 31]. A high density of H2Rs is found in the amygdala, hippocampus, cortex, and basal ganglia. Similarly, the action of H2Rs on neuronal membranes is also excitatory. The main pathway of H2Rs is the cAMP-PKA-CREB pathway, activating G*s* proteins and increasing cAMP formation, which leads to the regulation of neuronal plasticity [8, 15, 32].

**Fig. (1):**
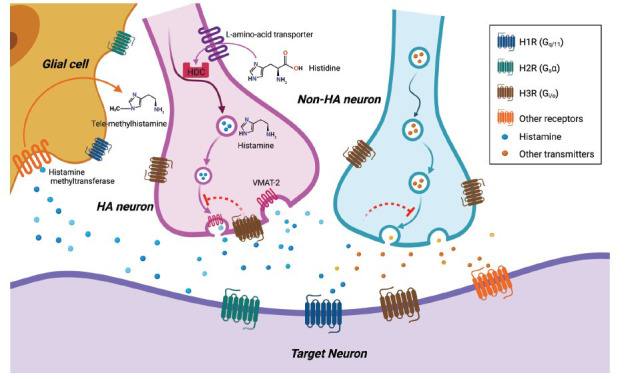
Central histaminergic signaling. Histidine is taken up *via* L-amino-acid transporter and decarboxylated by HDC to synthesis histamine; histamine is transported and released by VMAT-2, and is metabolized into tele-methylhistamine by histamine methyltransferase. H1Rs and H2Rs are located on post-synaptic membrane, while H3Rs are located on histaminergic and other cell somata, dendrites and axons (varicosities), where they provide feedback modulation to inhibit histamine and other transmitters synthesis and release. Postsynaptic targets of histamine neurons include somata and axon varicosities of many neurons and glial cells all over the CNS. **Abbreviations:** HA, histamine; VMAT-2, vesicular monoamine-transporter; HDC, histidine decarboxylase.

H3Rs are special and complex receptors among the four histamine receptors. In the brain, H1Rs and H2Rs only act as postsynaptic receptors and locate in neurons and glial cells. [33]. In contrast, H3Rs are only found in neurons. H3Rs act as presynaptic autoreceptors which regulate the release of histamine itself in histaminergic neurons [17, 34], as well as heteroreceptors in non-histaminergic neurons and regulate the release of diverse other neurotransmitters, including GABA, glutamate, acetylcholine and noradrenaline [35-37] (Fig. **1**). H3Rs are highly distributed in anterior parts of the cerebral cortex, hippocampus, amygdala, nucleus accumbens, striatum, olfactory tubercles, cerebellum, substantia nigra, and brain stem, mediating their functions through G*i/o* signaling [32, 38, 39].

H4Rs have been discovered recently and are predominantly expressed in the periphery tissues, such as bone marrow and leukocytes, but are also detected in the sensory dorsal ganglia, dorsal spinal cord, confined cortical and some thalamic regions of the brain [15, 18, 40]. Though similar in molecular homology and pharmacology to H3Rs [41], the therapeutic potential of H4R is still unclear currently and needs further study.

## 3 HISTAMINERGIC SYSTEM AND FEEDING BEHAVIOR

The notion that histamine might be a regulatory factor for feeding dates back to 1973 when Clineschmidt and Lotti first observed the inverse correlation between brain histamine level and appetite by administering histamine into the lateral ventricle of cats [42]. Later, plenty of consistent evidence supported brain histamine's role in regulating food intake and energy metabolism. Increasing brain histamine levels by intracerebroventricular infusion of histidine or H3R antagonists (*via* the function of presynaptic autoreceptors) suppress food intake [43-48]. Whereas sustained infusion of 𝛼-fluoromethyl histidine (𝛼-FMH, a suicide inhibitor of histidine decarboxylase) into the rat third cerebral ventricle, which decreases the brain histamine level, increases food intake [47, 49, 50]. Moreover, intracerebral infusion of histamine inhibited the development of obesity in both diet-induced and db/db obese mice in the long term [51]. Thus, it is for sure that histamine plays a critical role in feeding behavior; the underlying receptor-level mechanisms, however, remain to be elucidated.

### 3.1 Histamine Receptors and Feeding

Many studies have supported that H1Rs are essential for regulating food intake. H1R-KO mice exhibit increased daily food consumption and visceral adiposity [52]. For example, H1R-deficient mouse is a model of aging-related and diet-induced obesity. In addition, loading H1R-deficient mice with a high-fat diet increase fat deposition more than in wild mice [53]. Moreover, inhibition or depletion of H1Rs attenuates the anorexic effect induced by peripheral satiety signals, including leptin, amylin and nesfatin-1 [53-59]. While the third cerebral ventricle infusion of H3R inverse agonist thioperamide decreases food intake, the pretreatment with intraperitoneal (i.p.) injection of H1R antagonist chlorpheniramine abolishes this effect [47, 49]. Furthermore, the micro-infusion of H1R-antagonist pyrilamine into the suprachiasmatic nucleus attenuates the histamine-induced food intake suppression [60]. Contradictory to these observations, peripheral administration of H1R-antagonists mepyramine and chlorpheniramine does not increase food intake but decreases it [61, 62], suggesting the histaminergic system have a more complex work mode. Different modes of administration, the sensitivity or saturation state of drug targets, or different functions at the circuit level may cause conflict.

Although having a comparable amount of H1Rs in the CNS, H2Rs are deemed dispensable for feeding behaviors in previous studies. Lecklin and his colleagues found that systematic administration of H2R agonist dimaprit did not affect food intake [63]. Similarly, H2R-deficient mice showed normal food intake and body weight [64]. Interestingly, H2Rs may be more related to drinking behavior in previous studies. Injection of H1R and H2R antagonists abolished 60% of the food-related drinking by the rats, reduced the latency to drink after a meal and reduces drinking before a meal [65]. Furthermore, when administered centrally, the H2-receptor agonist amthamine or 4-methyl-HA stimulates arginine vasopressin secretion [66]. Blockade of H2 receptors abolishes the diuretic responses to histamine and dimaprit, suggesting that central H2Rs may mediate the diuretic effect of histamine [63]. A recent study suggested that deletion of H1R or H2R genes increases nonalcoholic fatty acid liver disease (NAFLD) in mice fed with a high-fat diet (HFD) [67]. Although data is lacking on although data is lacking on H2R involvement in feeding behavior in mice, cimetidine (an H2R antagonist) has been reported to suppress appetite and induce body weight loss in overweight adults [68, 69]. A recent study by Xu *et al.* revealed a novel medial septum (MS)-projecting histaminergic circuit regulating feeding behavior through downstream H2Rs on the glutamatergic (Glu) neurons in the MS [70]. Their data first identified the ability of the H2Rs in MS^Glu^ neurons to coordinate feeding behavior at the circuit level. Nevertheless, the direct correlation between H2Rs and feeding still lacks strong evidence resembling H1Rs and H3Rs.

H3Rs are also crucially involved in feeding behaviors. Thioperamide, an H3R inverse agonist, is reported to decrease food intake [63, 71]. Other novel H3R inverse agonists also display an ability to decrease food intake and body weight in long-term studies [72-74]. For example, the NNC38-1049 suppressed food intake and decreases body weight without changing the energy expenditure but reduces the respiratory quotient (RQ), indicating an increase in lipid oxidation [72]. However, in other studies, thioperamide did not affect food intake in satiated rats [75] or fasted [76]. These contradictory results may be caused by the complexity of the functions of H3 receptors as autoreceptors or heteroreceptors. In addition, Henry and his colleagues found that proxy fan (H3Rs protean agonist) reduces plasma glucose levels (300 mg/kg orally) by increasing plasma insulin levels and glucose excursion in a nongenetic type 2 diabetes mouse model (insulin sensibility not changed), which was not observed in H3R KO mice [77]. However, thioperamide (H3Rs inverse agonist) had a weaker effect compared to proxy an, while imetit (H3Rs agonist) did not affect glucose excursion [77]. This study further indicates the intricate work mode of H3Rs. H3R-KO mice are associated with behavioral state abnormalities, a metabolic syndrome with hyperphagia, late-onset obesity and increased levels of insulin and leptin [50, 78]. Although gene knockout of H1Rs and H3Rs can lead to obesity, the respective modes of their actions are unclear so far. Especially for H3Rs, whether it mediates its feeding-involved functions *via* histamine, non-histamine signaling, or other compensation factors remains unknown.

The H4R has a primary role in inflammatory functions, making it an attractive target for treating asthma and refractory inflammation [79]. However, the function of H4Rs in feeding is rarely studied so far.

The effects of gene knockout of the histamine receptors are summarized in Table **1**. The effects of pharmacological modulation of the histamine receptors are summarized in Table **2**.

### 3.2 Histamine-Specific Feeding Characteristics

The histaminergic system affects not only food intake but also feeding circadian rhythms. Several findings indicate that histamine may regulate feeding circadian rhythm through the hypothalamic H1Rs, and H1R-KO mice displayed disrupted diurnal feeding rhythms before the onset of metabolic syndromes and obesity [51, 52, 80]. This phenotype may be associated with the interference of circadian molecular clock genes and can be ameliorated by scheduled feeding [51, 81]. Furthermore, sustained infusion of 𝛼-FMH into the third cerebral ventricle disrupted light-dark feeding cycles in rats [11]. Besides, depletion of neuronal histamine by 𝛼-FMH enhances feeding-associated locomotor behavior only in the phase of the circadian cycle when histamine release is high [49].

Initially, brain histamine was believed to act as an appetite signal and mediated satiety [82, 83]. Later studies argued that brain histamine release is more related to motivated arousal (appetitive phases of feeding) than satiety signal (consummatory phases of feeding) [10, 84]. In addition, animal studies showed that that brain histamine is released to help rodents to maintain a state of high arousal before the anticipated meal, accompanied by increased locomotion [85-87]. Passani and his colleagues revealed that histamine release transiently increased when rats were trying to obtain food, while the histamine release did not change if rats had easy access to food [11]. Some important questions remain to be answered: (1) whether there is functional consistency with histamine release across different downstream projections during feeding; (2) whether endogenous histamine circadian rhythm (behavioral experiments performed in different time periods of the day) can affect the results of physiological function study; (3) whether brain histamine is also involved in drinking behavior and sodium appetite regulation except for food intake and if so, whether the physiological role of brain histamine is consistent in these ingestion-related behaviors?

### 3.3 Histamine Receptors as Targets for Feeding-Related Diseases and Current Challenges

From the therapeutic viewpoint, quite a few potential compounds targeted on H3Rs did not meet the expectations in clinical trials and were finally interrupted [11]. Unsatisfactorily, though proved to be involved in feeding, a number of H1Rs-related drugs failed to have the anti-obesity effects clinically, probably because they would activate peripheral H1R sites that could lead to severe cardiovascular, respiratory, or gastrointestinal side effects in the drug delivery process to the CNS [32]. Betahistine, a histamine analog with both H1R agonistic and H3R antagonistic effects, showed efficacy in preventing weight gain in schizophrenic patients using SGA drugs and females [88-90]. However, the clinical translation of some promising antagonists or agonists of H1Rs and H3Rs for treating feeding disorders is still challenging. On the one hand, further research should focus on developing brain-targeted histamine-related compound delivery to avoid peripheral adverse effects. The impacts of histamine-related compounds on weight control in main clinical studies are summarized in Table **3**.

On the other hand, one thing is for sure the histaminergic system has diverse receptors and innervations through CNS with different neural circuits. It is necessary to understand the mechanisms of the histaminergic network in regulating feeding from the circuit aspect, which is significant for the precise treatment of feeding-related diseases. From the perspective of the preclinical view, there are contradictory results of histamine-related pharmacological compounds and their effects on food intake. In many pharmacological studies on feeding, histaminergic ligands are often administered systematically, such as intracerebroventricular administration [50, 63, 77], intraperitoneal injection [62, 63, 91] and intragastric administration [92, 93]. However, intraperitoneal injection and intragastric administration cause unspecific bindings to peripheral histamine receptors, resulting in side effects such as affecting gastric acid secretion, to interfere with the treatment of feeding-related diseases. Although intraventricular administration may avoid these problems, it also affects the corresponding receptors in the whole brain without selectivity. Since neuronal histamine regulates brain function by binding to various receptors and is secreted by histaminergic neurons in TMN, which send projections to nearly all major brain regions, an important question is whether their modes of regulation are consistent in different projecting brain sites and neural circuits and whether the feeding-involved function of histamine receptors is consistent in different types of neurons. If not, the pathological changes of histamine receptor and histaminergic neural circuit might occur in specific parts instead of the whole brain under feeding-related pathological process; therefore, general administration might be inadvisable under all circumstances and could be the major account for the contrasting and frustrating results from preclinical and clinical studies.

## 4 HISTAMINE-MEDIATED FEEDING CIRCUITS: HOW DO HISTAMINERGIC NEURONS REGULATE FEEDING PRECISELY?

Initially, morphology studies showed that somata of histamine neurons are grouped within the TMN in five clusters (E1-E5), and there is no significant difference in the efferent connections between different compartments of TMN (medial and ventral subgroups) [94]. Recently, functional heterogeneity of the histaminergic neuron population has been recruited in a stressor- and subgroup-specific manner [95], suggesting the existence of differential expressions of functional neurotransmitter receptors across subgroups of these projecting neurons [96]. This is also supported by detecting *c-fos* after GSK189254 (an H3R antagonist) administration in cortical regions and TMN, but not in the striatum, in rats [97]. In line with these results, the infusion of thioperamide or GSK189254, but not bicuculline (a GABA_A_ receptor antagonist), into the TMN elicited histamine release in the rat prefrontal cortex and basal ganglia. This contrasts the histamine release caused by bicuculline, but not thioperamide or GSK189254, in the nucleus accumbens [98, 99]. These results indicated that the histaminergic network is organized in functionally distinct circuits impinging on different brain regions.

**Fig. (2):**
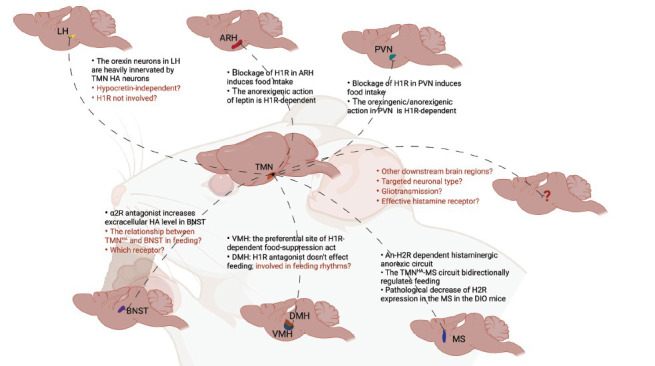
Possible histaminergic network on feeding regulation and unresolved questions. (**a**) The orexin neurons in the LH are heavily innervated by histaminergic axons [138] but mechanism of the cross-talk between these two neuron types are still unclear. (**b**) Administration of some potent H1R blockers in the ARH can increase food intake. This effect was attenuated by 𝛼-FMH [123, 124] and H1R-mediated excitation of the neurons in the ARC, which is a major site of leptin’s anorexigenic action [127]. (**c**) The H1R in the PVN can bidirectionally regulate feeding behavior [47, 49, 100, 102, 123, 166, 167]. (**d**) Yohimbine (an antagonist of 𝛼2 receptor) significantly increases the extracellular histamine content in the BNST [144] but the mechanism of the interaction between histaminergic neuron and the BNST neurons are largely unknown especially at the receptor level. (**e**) Bilaterally intra-VMH injection of H1R antagonist increases food intake, but it shows no response in the DMH site [49, 102] which may be involved in feeding rhythms regulation. (**f**) The H2R in the MS bidirectionally regulates feeding behavior and there is a pathological change of H2R expression in the MS in DIO mice [70]. (**g**) Other brain region innervated by the histaminergic neuron should be studied in feeding and the mechanisms need to be clarified at the cell type and receptor level. The known facts are listed in black and the unknown questions are addressed in red. **Abbreviations:** LH, lateral hypothalamus; ARH, arcuate nucleus of the hypothalamus; PVN, paraventricular nucleus; BNST, bed nucleus of the stria terminalis; VMH, ventromedial hypothalamus; DMH, dorsomedial hypothalamus; MS: medial septum; TMN, hypothalamic tubermammillary nucleus; DIO, diet-induced obesity.

Nevertheless, only a few studies have been conducted to investigate the relationship between feeding and histaminergic network in a circuit-specific way, conducted mainly by Sakata and his colleagues in the 1980s by stereotaxic administration [47, 49, 100-102] (Table **4**). Gratifyingly, using optogenetics, the precise non-histamine neural circuits in feeding have been well studied in recent years [103]. Several crucial nuclei have been proven involved in feeding, such as the paraventricular nucleus (PVN), the ventromedial hypothalamus (VMH), the dorsomedial hypothalamus (DMH), the arcuate nucleus of the hypothalamus (ARH), the lateral hypothalamus (LH), the bed nucleus of the stria terminalis (BNST), and more recently, the tuberal nucleus (TN) and zona incerta (ZI), *etc*. [104-108]. The crosstalk between these feeding-related nuclei and the histaminergic network is complicated and less understood (Fig. **2**). In the next part, we will interpret the underlying diverse histaminergic regulation mechanisms of feeding in a circuit-specific manner based on the above regions. However, most current results are not direct evidence in the circuit aspect. Thus, uncovering the precise circuitry phenotypes of the histaminergic system that orchestrates the feeding behavior is necessary.

### 4.1 Arcuate Nucleus of the Hypothalamus (ARH)

It is well recognized that the ARH plays a critical role in the regulation of feeding, as it fundamentally controls energy intake and metabolism. ARH mainly contains two sets of neurons; one is orexigenic (appetite-inducing) AgRP (NPY/GABA) neurons, and the other one is anorexigenic (appetite suppressing) POMC/CART neurons, which respond to energy signals (*e.g*., adipose-released hormone, leptin), GI-derived satiety signals (*e.g*., CCK), and food deprivation (ghrelin) [109, 110]. AgRP neurons are necessary and sufficient for mediating feeding behavior: both chemogenetic inhibition and genetic ablation of AgRP neurons significantly decreased feeding and lead to rapid starvation in adults [111, 112]; in contrast, optogenetic or chemogenetic activation of AgRP neurons causes a rapid and reversible increase in food intake [111, 113], probably through bypassing the effects of satiety signals and induce feeding by restoring hunger-like patterns of activity in the insular cortex [114]. In contrast, activating POMC neurons optogenetically or chemogenetically, which can also be activated by leptin [115], decreased food intake [113, 116]. Activation of ARC^POMC^-PVN evoked feeding requires inhibition of PVN^MC4R^ neurons [117]. More recent optogenetics studies found that activation of AgRP neurons induces feeding to overcome the appetite-suppressing effects triggered by amylin, cholecystokinin (CCK), and lithium chloride (LiCl) [118]. Moreover, low-frequency stimulation of Kiss1 neurons in ARH directly excited POMC and AgRP neurons *via* glutamate release, leading to motivational feeding behavior [119]. Until now, we still cannot compete for the importance of these two sets of neurons in ARC. However, Wei and his colleagues recently found that simultaneous stimulation of both POMC neurons and a subset of the orexigenic AgRP neurons is sufficient to reverse that inhibition and trigger intense feeding behavior [120], suggesting the activation mechanisms might be more translational than the inhibition mechanisms in controlling appetite.

Key sites known to mediate food intake and energy balance include DMH, PVH, and LH [121]. The ARH is also innervated by histaminergic neurons [2]. It was observed that atypical antipsychotic drugs (AAPDs) stimulate appetite and induce weight gain through selective activation of hypothalamic AMP kinase, linked to food intake regulation [122], and thus reverse the actions of the anorexigenic hormone leptin. These drugs were proved to be potent H1R blockers by binding assays [123], which could be attenuated by pretreatment with 𝛼-FMH [124]. Thus, the increasing food intake may also result from the blockage of H1Rs. Moreover, AAPD augmentation of appetite was abolished in mice with the deletion of H1 receptors [125]. The interaction between the histaminergic system and leptin-induced suppression of food intake is evidenced by using H1R-KO mice [126]. And the ARH is a major site of leptin’s actions, which is, in turn, required for the normal development of ARH pathways. Tuomisto *et al.* also found that H1R-mediated excitation of the neurons in the ARC responsive to substance P [127]. These interesting findings suggest that the histaminergic innervation in ARH might likely play a role in feeding regulation in leptin- and H1R- dependent manner, which needs further study to illustrate the underlying mechanism.

### 4.2 Lateral Hypothalamus (LH)

The LH is a well-known feeding center that regulates appetite and hunger [105]. It is composed of heterogeneous neural populations. The LH mainly contains two types of neurons; one is GABAergic neurons, and the other one is glutamatergic neurons. Like the ARC, LH neurons express receptors for hormones and factors signaling energy status, such as orexin/hypocretin (Hcrt), melanin-concentrating hormone (MCH), leptin receptor (LepR), -expressing neurons. Optogenetic stimulation of LH^GABA^-PVH pathways can evoke feeding behavior [128], whereas inhibiting this circuit reduces feeding after fasting. Moreover, inhibition of neurotransmission in the LH vesicular glutamate transporter (VGLUT2) positive neuron terminals in the lateral habenula (LHb) promotes feeding behavior [129]. Another study showed that inhibition of LH GABAergic fibers in the PVH reduces feeding in fasted mice [130].

Apart from receiving multiple excitatory and inhibitory inputs from both cortical and subcortical structures [131], LH also receives inputs from TMN [2, 132]. There are indirect trans-synaptic regulations between LH and TMN. For instance, the LH is innervated by inhibitory GABAergic subcortical fibers from the lateral septum and much of the basal forebrain, which receive strong signals projecting from TMN [8, 133]. Neuromodulators, including histamine [134, 135], dopamine, norepinephrine [136] and serotonin [137], are also released in the LH, where they can act to sculpt circuit dynamics further. As mentioned above, the hypothalamic Hcrt-neurons, one of the main neuron groups in the LH, are associated with regulating sleep and feeding [110], consistent with the role of histamine in regulating sleep and feeding [8]. Immunocytochemical studies have shown that the histaminergic and orexin neurons often locate near [138]. The interplay between these two types of neurons seems reciprocal because histaminergic axons heavily innervate the orexin neurons in TMN. These results suggest a functional connection between the two populations of hypothalamic Hcrt-neurons and histaminergic neurons. Therefore, we speculate that they may cooperate in regulating sleep and feeding in some aspects.

Although there is a strong mutual innervation and functional interaction between these two neuron groups, *in vitro* electrophysiological records only revealed one direction: hypocretins excite histaminergic neurons [138]. However, histamine does not affect the spike frequency and evokes outward currents of Hcrt-neurons [139]. This one-way effect suggests that the TMN-LH circuit regulates feeding, if any, probably *via* a hypocretin-independent manner. On the contrary, pharmacological studies showed that injecting H1R antagonists into LH or other hypothalamic nuclei does not induce feeding in rats [100, 102]. In light of these findings, other neurotransmitters and receptors should be investigated in moderation for feeding *via* the TMN output to the LH.

### 4.3 The Bed Nucleus of the Stria Terminalis (BNST)

The BNST is a heterogeneous and complex limbic forebrain structure that plays a vital role in regulating anxiety and is well-recognized for its function in feeding recently [140]. The BNST has diverse cell subpopulations, such as corticotropin-releasing (CRH) and protein kinase C-𝛿 (PKC-𝛿) neurons. Chemogenetic inhibition of PKC-𝛿 neurons in BNST can effectively attenuate inflammation-associated anorexia [106]. Activating the inhibitory GABAergic nerve terminals in BNST projected from ARC AGRP neurons or somatostatin neurons in the tuberal nucleus (TN) increases food intake [108, 141, 142]. Direct optogenetic stimulation of the vBNST^GABA^-LH^Vglut2^ circuit produces robust feeding behavior correlated with stimulation frequency [143] and directed toward the palatable, calorie-dense foods available. In contrast, optogenetic inhibition of this circuit reduces feeding in food-deprived mice [143].

Yohimbine, an antagonist of 𝛼2 receptor, significantly increased the extracellular histamine content in the BNST [144]. Nevertheless, the interaction of BNST neurons and histaminergic neurons in feeding remains unexplored. Whether histamine activity in BNST can affect feeding behavior is still a question that needs further studies to uncover the answer.

### 4.4 Ventromedial and Dorsomedial Hypothalamus (VMH/ DMH)

The VMH and DMH are other crucial hypothalamic sites in feeding behavior [145]. The notion that VMH neurons are important in body weight regulation came from early studies, which have shown that lesions in this area caused marked obesity and overfeeding, suggesting that neurons in this region limit excessive food intake [146]. The VMH is also composed of several diverse neuron sets, one representative, and the well-studied population is recognized as SF-1 neurons (SF-1, steroidogenic factor-1) [147]. However, DMH action on feeding is thought to be more relevant to circadian aspects of food intake [148], as lesions cause disruptions in the feeding rhythms [149]. Moreover, cell-type specific manipulation in recent studies has shown that projections from GABAergic DMH^LepR^ neurons to ARC^AgRP^ neurons were reported to suppress feeding [150], whereas the DMH^GABA^-PVN circuit is promoted feeding [151]. Additionally, cholinergic neurons in the DMH increase feeding by enhancing GABAergic neurotransmission onto ARC^POMC^ neurons [152]. Thus, these two sites act on feeding through diverse cell types and circuits.

Neurons in the VMH and DMH receive input from the ARC and the suprachiasmatic nucleus (SCN) and regulate feeding rhythms [148, 153]. Injecting H1R antagonists in the VMH, but not PVN or LH, effectively regulated appetite [100], suggesting that the VMH is likely the preferential site of histamine-mediated suppression of food intake. In keeping with these results, electrophysiological records have shown suppression of the firing of glucose-responsive units in the VMH but not LH or PVN after applying H1R antagonists [154]. The DMH, on the other hand, conveys circadian-photic and nutritional-metabolic influences from the SCN and ARC, which is crucial for a wide range of behavioral circadian rhythms [155]. In addition, the DMH is also innervated by the histaminergic neurons and is known to be involved in the regulation of food intake [8, 156]. Specifically, DMH lesions produced hyperphagia and weight loss [156]. These studies emphasize the convergence of circadian, histaminergic, and hypocretin systems in synchronizing neural activities and molecular clockwork in the DMH [8]. However, while bilaterally, intra-VMH injection of chlorpheniramine, an H1R antagonist, increases food intake, it shows no response in the DMH site [49, 102].

Therefore, the TMN^HA^-VMH circuit may play a more important role in the regulation of feeding, while the TMN^HA^-DMH circuit needs further studies to reveal its function in feeding, probably in the rhythms-related feeding aspect.

### 4.5 Paraventricular Nucleus (PVN)

The PVN is a heterogenous hypothalamic region that contains a diverse of non-peptidergic and peptidergic-expressing neurons, including oxytocin-, vasopressin, thyrotropin-releasing hormone (TRH), corticotropin-releasing hormone (CRH) and pituitary adenylate cyclase-activating polypeptide (PACAP, also known as ADCYAP1) neurons [157]. Lesions of the PVN produce hyperphagia and obesity, demonstrating the significance of PVN neurons in metabolism and feeding regulation [158-160]. It was later discovered that PVN neurons abundantly express melanocortin receptor 4 (MC4Rs) [117, 161], whose inefficiency will lead to early-onset obesity both in mice and humans [162, 163]. Some research indicated that excitation of PVN^MC4R^-LPBN and PVN^Sim1^-PAG/DR can reduce feeding [117, 164]. In addition, the PVN sends strong excitatory inputs to the ARH, specifically from subsets of TRH and PACAP neurons. Chemogenetic stimulation of these afferent neurons in the ARH in sated mice markedly activates ARH^AgRP^ neurons and induces intense feeding [165].

Several lines of evidence suggest that histamine can decrease food intake *via* H1Rs in the PVN, which is one of the richest areas of hypothalamic histamine and H1Rs [47, 49, 100, 102]. Based on some findings, it is evident that the histamine and H1Rs in PVN are involved in antidepressant- and antipsychotics-induced food intake. The i.p. injections of antihistaminic antidepressants doxepin [166] and promazine [167] significantly increase food intake. And amitriptyline, which is a potent H1-blocker, elicits food intake when infused into the third cerebral ventricle and PVN bilaterally [123]. Nevertheless, haloperidol and desipramine, relatively weak H1R-antagonists [123], did not affect food intake [167], suggesting that the TMNHA-PVN circuit may regulate feeding in a dose-dependent way.

In addition, the effects of histamine on food intake were associated with several other neuroendocrine and peptidergic pathways [51, 126]. Orexigenic actions of orexins/hypocretins and anorexigenic effects of leptin and glucagon-like peptide-1 (GLP-1) depend on Hcrt released by PVN neurons; all abolished in H1R-deficient or H1R-KO mice [168-170].

Based on these findings, it is concluded that H1Rs in PVN are crucial receptors for regulating feeding in the TMNHA-PVN circuit. Many other pathways can be looked into to further elucidate the TMN projecting fibers' function in PVN feeding behavior.

### 4.6 Medial Septum (MS)

The MS is a newly recognized nucleus that plays a vital part in controlling feeding behavior. Previous studies have identified three neuron types in the MS, that is, cholinergic, GABAergic and glutamatergic neurons, which are all involved in feeding regulation [171-174]. Specifically, inhibition of all three neuron types in the septal region consistently induces feeding behavior and *vice versa*. Interestingly, using the fluorescence micro-optical sectioning tomography (fMOST) system, our unpublished anatomical data indicate that the MS receives a relatively high density of histaminergic projections from the TMN. This anatomical connection raises the possibility that an MS-projection histaminergic circuit may be involved in feeding behavior, which excite our interest.

As expected, our recent study found that the TMN^HA^-MS circuit does participate in regulating feeding behavior; selective inhibition of the TMN-MS histaminergic circuit drives food consumption, while activation of this circuit can suppress food consumption, suggesting the MS is an important targeted region that is associated with histamine-modulated feeding behavior. Even more surprisingly, this circuit mediates feeding *via* downstream H2Rs rather than H1Rs, which has been proved to be more related to feeding in previous studies [70]. Importantly, we found a pathological decrease of H2R mRNA expression in MS^Glu^ neurons and downregulation of the H2R expression using shRNA interference significantly accelerates the body weight gain in high-fat diet (HFD)-fed mice. In addition, chronically activating the MS^Glu^ neurons *via* H2Rs agonist amthamine could significantly reduce the body weight gain in diet-induced obesity (DIO) mice. This study gives insight into potential targets for treating feeding-related disorders and sets a precedent for exploring other specific histaminergic circuits regulating feeding behavior.

## CONCLUSION AND REMARKS

A horizontal understanding of molecular, cellular, and circuit levels is conducive to accurate regulation and treatment for feeding-related diseases. Certainly, in the past decades, much has been learned about the role of histamine as a neurotransmitter in feeding [8, 96, 175]. The availability of histamine-related tool drugs helped scientists dissect histamine receptors' functions separately, mainly in a general way with systematic administration. Summarizing the complex findings is necessary to dissect histamine-related feeding behavior mechanisms precisely, especially at the circuit level. To date, accurate expertise on cellular, circuit and brain region levels regarding the relationship between histaminergic network and feeding is just at the infant stage. How histaminergic neurons in these canonical circuits communicate with other neurons and coordinate to regulate the complex feeding behavior is still poorly understood (Fig. **2**). And here, we provide three potential research interests in this field: (1) understanding the different brain histaminergic substrates mediating different characteristics of feeding behavior (*e.g*., motivational “wanting” and hedonic “liking” for food); (2) elucidating the links between the functions of peripheral histamine, central histaminergic network (including astrocytes), gut-brain circuits and feeding behavior; (3) developing ways of histamine-related drugs administration for precise treatment for eating disorders.

Fortunately, the development of optogenetics [176], viral tracing [177, 178] and CLARITY techniques [179] combined with the Cre-Loxp strategy (the transgenetic *HDC-CreERT2* mouse line and *HDC-Cre* rat line are available now [180, 181]), has enabled us to clarify the structure and functions of histaminergic cells in neural circuit level in a more precise way. For example, the fluorescence micro-optical sectioning tomography (fMOST) system is an appropriate tool for analyzing the complex structure of a histaminergic network, which may provide some orderly breakthroughs for studying specific functional circuits. With an acute whole-brain mapping of the histaminergic neurons in the mouse brain, we can: (1) sort out the potentially valuable downstream projections with the projection patterns of histaminergic neurons; (2) dissect the relationship between the density of downstream histaminergic nerve fibers and its function; (3) investigate the pathological changes, if any, of the projection patterns of histaminergic neurons in different eating disorder models. At present, optogenetics is beginning to reveal the functional circuitry of histaminergic neurons in several behaviors, such as sleeping [182] and obsessive-compulsive-like behaviors [181], and we believe that it can also be finely applied to the feeding field.

Recently, using optogenetics, we found that the TMN^HA^-MS circuit did participate in regulating feeding behavior, and selective modulation of the TMN-MS histaminergic circuit bi-directionally drives food consumption [70]. Previous pharmacological studies of brain region administration have laid a good foundation for the follow-up work in the histaminergic circuits of feeding behavior. Notably, apart from the MS and the aforementioned nuclei, other downstream nuclei of the TMN histaminergic neurons may also involve in the feeding behavior. For example, recent studies have shown that the PAG contributes to appetitive behavior [183]. Interestingly, some early studies showed that the PAG expressed a relatively high number of histaminergic receptors [184]. H2R activation in the PAG was reported to be involved in defensive behavior [185] and may be more related to the dPAG region [186, 187]. In addition, histamine in the PAG could induce antinociception [188, 189]. Nevertheless, the underlying mechanism of action between histaminergic neurons and the PAG neurons is largely unexplored in feeding behavior, which is worthwhile to investigate in a future study.

On the other hand, the function of cell-specific histamine receptors in feeding is another crucial aspect for future acute treatment. Recent studies have shown that histamine receptors possess cell-specific functions in different cell types [190, 191]; thus, it is important to dissect the particular functions of these cell-specific receptors in the neuronal circuits. Notably, the role of astrocytes in regulating feeding should be considered, which is relatively neglected for now. Previous studies have reported the expression of H1/H2 receptors on astrocytes [192, 193]. With a similar binding capacity for histamine compared to neurons, astrocytes could be one of the main targets of the histaminergic system in the brain [194, 195]. Histamine stimulation increases [Ca^2+^] _I_ in astrocytes [196, 197]. Furthermore, a connection between histamine treatment and enhancing glutamate release from astrocytes *via* H1Rs was reported [198]. Only a few studies to date, however, have investigated the effect of histamine on gliotransmitter release, which is also important for us to understand the comprehensive mechanisms underlying eating disorders. Using of *Hrh1-3R^fl/fl^* transgenic animals and specific Hrh1-3 knockout or silencing virus tools is a potential way to solve the above problems, and such experiments have been performed in the studies of histamine receptor functions in several behaviors or diseases [199, 200], which should also apply to the study of feeding.

Up to now, much experimental evidence suggests that the histaminergic system is organized into distinct pathways and modulated by selective mechanisms [95, 97-99]. This could imply different working modes of subsets of histaminergicneurons according to their projection fibers and downstream receptors. Consequences could be relevant for understanding the precise mechanisms of diverse circuits, thus increasing the drug specificity and the efficiency of treating eating disorders.

## Figures and Tables

**Table 1:** Impacts of genetically knockout histamine-related genes on feeding.

**Animal Model**	**Phenotype on Feeding**	**References**
H1R-KO mice	Diet-induced and aging-related obesity; disturbed feeding rhythms; increase in daily food consumption; hyperphagia and decreased expression of UCP-1 mRNA; attenuated anorectic effect of leptin	[52, 53, 56]
H3R-KO mice	Hyperphagia; late-onset obesity associated with hyperinsulinemia and leptinemia	[50, 78, 201]
HDC-KO mice	Hyperleptinemia; visceral adiposity; decreased glucose tolerance; increased susceptibility to HFDIO	[202-204]

**Note**: HFDIO, high-fat diet-induced obesity; UCP-1, uncoupling protein-1; —, no obvious evidence so far.

**Table 2:** Effects of histaminergic drugs on feeding behavior.

**Compounds**	**Drug Actions**	**Mode of Administration**	**Main Findings**	**References**
**H3R-Relataed Compounds**				
Thioperamide	H3R inverse agonist	ICV, 100 nM	Food intake ↓	[47]
		ICV, 100 nM	Food intake ↓	[49]
		ICV, 200 nM	Prevent H3R agonist-induced drinking behavior	[63]
		i.p., 3 mg /kg	Food intake ↓	[205]
		p.o., 5 ml/kg; ICV, (500 nM, 1 μl per head)	Food intake ↑, body weight ↑	[50]
Imetit	H3R agonist	i.p., 20 mg∙kg^-1^	Food intake ↓, body weight ↓	[50]
(R)α-methylhistamine (Rα-MeHA)	H3R agonist	ICV, 100 nM	Drinking behavior ↑	[63]
		i.p., 0.3-3 mg /kg	Food intake —, but reversed the thioperamide-induced inhibition of food intake in a dose-dependent manner.	[205]
NNC38-1049	H3R inverse agonist	p.o., 60 mg/kg; i.p. 20 mg/kg	Food intake ↓, body weight ↓, water intake ↓ (short term); — (long term).	[72]
NNC 38-1202	H3R inverse agonist	rats (5 mg/kg p.o. for 22 days)/ pig (5/15 mg/kg intra gastric)/ monkey (0.1/1 mg/kg s.c.)	Rats: food intake ↓, body weight ↓ (HFD model); Pig: food intake ↓; Monkey: food intake ↓	[73] [74]
(4,4-Difluoroeperidin-1-yl)[1-isopropyl-5-(1-isopropyliperidin-4-yloxy)-1H-indol-2-yl]methanone	H3R inverse agonist	p.o., 5/10/20 mg/kg	Food intake ↓, and reversed the (R)-α-methylhistamine-induced water intake	[93]
A-331440	H3R inverse agonist	p.o., 0.5/5/15 mg/kg	0.5 mg/kg: no effect on weight 5 mg/kg: food intake ↓, body weight ↓ 15 mg/kg: body weight ↓↓	[92]
A-423579	H3R inverse agonist	p.o., 3/10 mg/kg	Body weight ↓, food intake↓ No effect on oxygen consumption	[206]
A-417022	H3R inverse agonist	p.o., 10/30 mg/kg	10 mg/kg: no effect 30 mg/kg: body weight ↓ (comparable to A331440)	[207]
A-631972	H3R inverse agonist	p.o., 0.5/1.5 mg/kg	No effect	[206]
**H1R-Relataed Compounds**				
Chlorpheniramine	H1R antagonist	ICV 0.26 μM	Food intake ↑ (early light); Food intake — (early dark)	[100]
		i.p., 1/5/10 mg/kg	Food intake ↓(10 mg/kg) Water intake: ↓ (10 mg/kg)	[62]
2-(3-trifuloromethylphenyl) histamine (FMPH)	H1R agonist	i.p. 1/5/10 mg/kg	Food intake: ↓	[63]
Cetirizine	H1R antagonist	p.o., ~4 mg/kg (water containing cetirizine 20 mg/l)	Food intake — (inability to cross BBB?) Body weight ↑	[91]
**H2R-Relataed Compounds**				
Cimetidine	H2R antagonist	ICV, 0.48 μM	Food intake —	[154]
Dimaprit	H2R agonist	ICV, 100 nmol, 5-10 μl	Long-lasting diuresis	[63]

**Note:** ICV, intra-cerebroventricular injection; i.p., intraperitoneal injection; p.o., peros; s.c., subcutaneous injection.

**Table 3:** Effects of histamine-related drugs in main clinical studies.

**Compounds**	**Target**	**Subjects**	**Trial ** **Duration**	**Treatment**	**Main Findings**	**References**
Betahistine	H1R & H3R	Healthy females (n=46) 18-45 years 16.8 < BMI < 27	4 weeks	Day 1-7: Placebo (n=22); Betahistine (144 mg/day) (n=24) Day 8-14: Placebo + Olan (n=22); Betahistine (144 mg/day) + Olan (n=24) Day 15-28: Olan only for two groups	Less ∆BW compared to the placebo groups (*p*<0.05)	[89]
		Diagnosed with schizophrenia or bipolar disorder (17 females + 25 males) 18-55 years BMI = 25.23 ± 2.33	12 weeks	Placebo + SGA (n=29) Betahistine (36 mg/day) + SGA (n=13)	Less ∆BW compared to the placebo groups (*p*<0.05); Less ∆BMI compared to the placebo groups (*p*<0.05)	[208]
		Obese adults (n=234, females and males) 18-65 years 30 < BMI < 40	12 weeks	Placebo (n=63) Betahistine (16 mg/day) (n=55) Betahistine (32 mg/day) (n=58) Betahistine (48 mg/day) (n=58)	No significant weight loss at the doses tested; while subgroup analysis revealed that betahistine induced significant weight loss only in females below 50 years group (*p*=0.05)	[90]
Ranitidine	H2R	Diagnosed with a first episode of schizophrenic disorder (8 females and 67 males) 18-60 years BMI < 30	8 weeks	Placebo + Olan (n=25) Ranitidine (150 mg/day) + Olan (n=25) Ranitidine (300 mg/day) + Olan (n=25)	no significance at the tested doses compared with the indicated group.	[209]
Famotidine	H2R	Hospitalized for a first episode of acute psychosis (5 females and 9 males) 40-65 years BMI < 30	6 weeks	Placebo+ Olan (n=7) Famotidine (40 mg/day) + Olan (n=7)	no significance at the tested doses compared with the indicated group.	[210]
Nizatidine	H2R	Diagnosed with schizophrenia (14 females and 21 males) 28.7 ± 8.8 years BMI= 26.8 ± 1.7	8 weeks	Placebo + Olan (n=17) Nizatidine (300 mg/day) + Olan (n=18)	Less BW compared to the baseline (*p* < 0.05); Less ∆BW compared to the placebo groups (*p* < 0.05); Less BMI compared to the baseline (*p* < 0.05); Less ∆BMI compared to the placebo groups (*p* < 0.05)	[211]
		Diagnosed with schizophrenia, schizoaffective disorder, or schizophreniform disorder (22 females and 32 males) 18-65 years BMI < 40	12 weeks	Placebo + Olan (n=27) Nizatidine (300 mg/day) + Olan (n=27)	no significance at the tested doses compared with the indicated group.	[212]
		Diagnosed with schizophrenia, schizoaffective disorder, or schizophreniform disorder (n=169) 18-65 years BMI < 40	16 weeks	Placebo + Olan (n=56) Nizatidine (300 mg/day) + Olan (n=56) Nizatidine (600 mg/day) + Olan (n=57)	no significance at the tested doses compared with the indicated group.	[213]
Cimetidine	H2R	Overweight adults with type 2 diabetes (14 females and 29 males) 18-65 years 27.2 < BMI < 48.2	12 weeks	Placebo (n=24) Cimetidine (1200 mg/day) (n=19)	Less BW compared to the baseline (*p* < 0.05); Less ∆BW compared to the placebo groups (*p* < 0.05); Less BMI compared to the baseline (*p* < 0.05);	[69]
		Overweight adults (55 females and 5 males) 18-59 yrats 25 < BMI < 37	8 weeks	Placebo (n=30) Cimetidine (600 mg/day) (n=30)	Less ∆BW compared to the placebo groups (*p* < 0.001); Less ∆BMI compared to the placebo groups (*p* < 0.001)	[68]

**Abbreviations:** SGA, second-generation antipsychotic drugs; BW, body weight; BMI, body mass index; ∆BW, change of body weight; ∆BMI, change of body mass index; Olan, olanzapine.

**Table 4:** Effects of brain local administration of histaminergic ligands on feeding behavior.

**Regions**	**Compounds**	**Mode of Administration**	**Main Findings**	**References**
VMH	α-FMH	Bilaterally micro-infusion (224 nM)	Food intake ↓	[47]
	Chlorpheniramine	Bilaterally micro-infusion (maximal dose of 26 nM)	VMH is the main locus for the induction of feeding by chlorpheniramine, the effect was abolished when pretreated with α-FMH	[100]
		Bilaterally micro-infusion (maximal dose of 26 nM)	Food intake ↑ (the early light)	[101]
		Bilateral micro-infusion (maximal dose of 26 nM) (unilateral infusion did not work)	Food intake ↑	[102]
		Bilaterally micro-infusion (maximal dose of 52 nM)	Food intake ↑	[49]
	H2R antagonists (Unknown)	Bilaterally micro-infusion	—	[49, 102]
PVN	α-FMH	Bilaterally micro-infusion (224 nM)	Food intake ↓	[47]
	Chlorpheniramine	Bilaterally micro-infusion (maximal dose of 26 nM)	PVN is not the main locus for the induction of feeding by chlorpheniramine	[100]
		Bilaterally micro-infusion (maximal dose of 26 nM)	—	[101]
		Bilaterally micro-infusion (only 52 nmol works)	Food intake ↑	[102]
		Bilaterally micro-infusion (52 nM)	Food intake ↑	[49]
	H2R antagonists (Unknown)	Bilaterally micro-infusion	—	[49]
LH	α-FMH	Bilaterally micro-infusion (224 nM)	—	[47]
	Chlorpheniramine	Bilaterally micro-infusion (maximal dose of 26 nM)	LH is not the main locus for the induction of feeding by chlorpheniramine	[100]
		Bilaterally micro-infusion (maximal dose of 26 nM)	—	[101]
		Bilaterally micro-infusion (maximal dose of 52 nmol)	—	[102]
		Bilaterally micro-infusion (maximal dose of 52 nmol)	—	[49]
	H2R antagonists (Unknown)	Bilaterally micro-infusion	—	[49]
DMH	α-FMH	Bilaterally micro-infusion (224 nM)	—	[47]
	Chlorpheniramine	Bilaterally micro-infusion (maximal dose of 52 nmol)	—	[102]
		Bilaterally micro-infusion (maximal dose of 52 nmol)	—	[49]
	H2R antagonists (Unknown)	Bilaterally micro-infusion	—	[49, 102]
POAH	α-FMH	Bilaterally micro-infusion (224 nM)	—	[47]
	Chlorpheniramine	Bilaterally micro-infusion (maximal dose of 52 nmol)	—	[102]
		Bilaterally micro-infusion (maximal dose of 52 nmol)	—	[49]
	H2R antagonists (Unknown)	Bilaterally micro-infusion	—	[49]
MS	Thioperamide (H3R inverse agonist)	Micro-infusion (3 μM, 500 nl)	Food intake ↓	[70]
	Pyrilamine (H1R antagonist)	Micro-infusion (10 μM, 500 nl)	—	[70]
	Amthamine (H2R agonist)	Micro-infusion (10 μM, 500 nl)	Food intake ↓	[70]

**Note**: VMH, ventromedial nucleus of hypothalamus; PVN, paraventricular nucleus; LH, lateral hypothalamus; DMH, dorsomedial nucleus of hypothalamus; POAH, preoptic anterior hypothalamus; MS, medial septum; —, no effect.

## LIST OF ABBREVIATIONS

BNST: Bed Nucleus of the Stria Terminalis
DMH: Dorsomedial Hypothalamus
HFD: High-Fat Diet
LH: Lateral Hypothalamus
NAFLD: Nonalcoholic Fatty Acid Liver Disease
PVN: Paraventricular Nucleus
RQ: Respiratory Quotient
SCN: Suprachiasmatic Nucleus
TN: Tuberal Nucleus
VMH: Ventromedial Hypothalamus
ZI: Zona Incerta
